# The prolyl 4-hydroxylase inhibitor GSK360A decreases post-stroke brain injury and sensory, motor, and cognitive behavioral deficits

**DOI:** 10.1371/journal.pone.0184049

**Published:** 2017-09-07

**Authors:** Jin Zhou, Jie Li, Daniel M. Rosenbaum, Jian Zhuang, Carrie Poon, Pu Qin, Katrina Rivera, John Lepore, Robert N. Willette, Erding Hu, Frank C. Barone

**Affiliations:** 1 Department of Neurology, State University of New York Downstate Medical Center, Brooklyn, New York, United States of America; 2 Robert F. Furchgott Foundation, State University of New York Downstate Medical Center, Brooklyn, New York, United States of America; 3 Department of Physiology and Pharmacology, State University of New York Downstate Medical Center, Brooklyn, New York, United States of America; 4 Cardiac Biology, Heart Failure Discovery Performance Unit, GlaxoSmithKline Pharmaceuticals, King of Prussia, Pennsylvania, United States of America; University of South Florida, UNITED STATES

## Abstract

There is interest in pharmacologic preconditioning for end-organ protection by targeting the HIF system. This can be accomplished by inhibition of prolyl 4-hydroxylase (PHD). GSK360A is an orally active PHD inhibitor that has been previously shown to protect the failing heart. We hypothesized that PHD inhibition can also protect the brain from injuries and resulting behavioral deficits that can occur as a result of surgery. Thus, our goal was to investigate the effect of pre-stroke surgery brain protection using a verified GSK360A PHD inhibition paradigm on post-stroke surgery outcomes. Vehicle or an established protective dose (30 mg/kg, p.o.) of GSK360A was administered to male Sprague-Dawley rats. Initially, GSK360A pharmacokinetics and organ distribution were determined, and then PHD-HIF pharmacodynamic markers were measured (i.e., to validate the pharmacological effects of the GSK360A administration regimen). Results obtained using this validated PHD dose-regimen indicated significant improvement by GSK360A (30mg/kg); administered at 18 and 5 hours prior to transient middle cerebral artery occlusion (stroke). GSK360A exposure and plasma, kidney and brain HIF-PHD pharmacodynamics endpoints (e.g., erythropoietin; EPO and Vascular Endothelial Growth Factor; VEGF) were measured. GSK360A provided rapid exposure in plasma (7734 ng/ml), kidney (45–52% of plasma level) and brain (1–4% of plasma level), and increased kidney EPO mRNA (80-fold) and brain VEGF mRNA (2-fold). We also observed that GSK360A increased plasma EPO (300-fold) and VEGF (2-fold). Further assessments indicated that GSK360A reduced post-stroke surgery neurological deficits (47–64%), cognitive dysfunction (60–75%) and brain infarction (30%) 4 weeks later. Thus, PHD inhibition using GSK360A pretreatment produced long-term post-stroke brain protection and improved behavioral functioning. These data support PHD inhibition, specifically by GSK360A, as a potential strategy for pre-surgical use to reduce brain injury and functional decline due to surgery-related cerebral injury.

## Introduction

A short duration of ischemia (i.e., ischemic preconditioning; IP) can provide significant brain protection to subsequent long-duration ischemia (i.e., termed ischemic tolerance; IT). Thus, injurious stimuli applied to an organ below its injury threshold can activate endogenous protective mechanisms that involve newly expressed protective proteins [1–9]. IP induced IT has been shown in the human nervous system and heart [10, 11] suggesting that we can capture these endogenous neuroprotective mechanisms for use to protect the brain and heart in surgical patients. A major mechanism for IP-induced IT is signaling that occurs via the increased activity of hypoxia inducible factor (HIF), a master regulator of oxygen homeostasis. For example, HIF-1α is required for IP, and a partial HIF-1α deficiency in heterozygote knockout mice leads to complete loss of IP-induced cardioprotection [12]. The breadth and depth of inducing this “IP-like” protective signaling has been suggested to offer new opportunities for stroke therapeutic intervention in the face of much pessimism over many failed clinical stroke trials [13, 14]. In any event, the HIF signaling pathway is now well-defined and provides opportunities to use chemical agents/drugs that stimulate this endogenous protective pathway.

**Fig 1:**
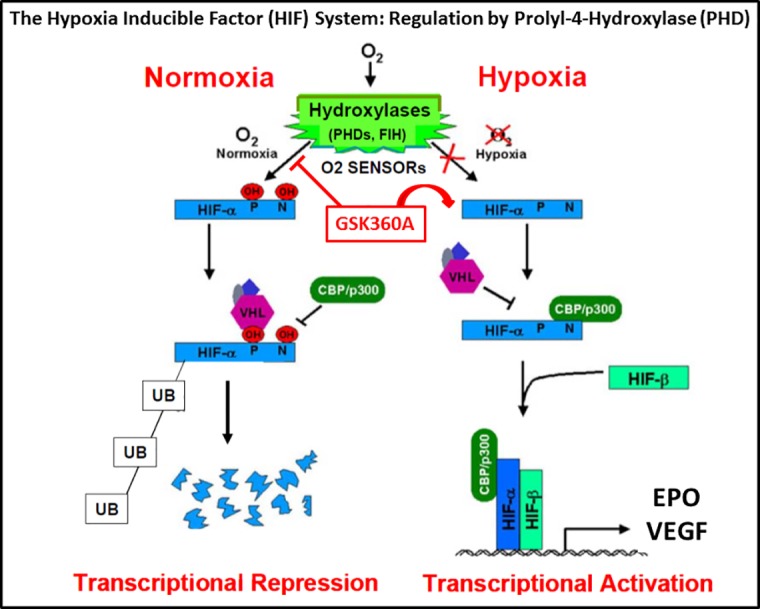
Hypoxia-inducible factors (HIFs) are transcription factors that respond to changes in environmental oxygen. Three HIF prolyl-4-hydroxylases (PHDs) and one HIF asparaginyl hydroxylase (factor inhibiting HIF; FIH) act as oxygen (O2) sensors by regulating the hydroxylation of HIF-α, which controls the stability and transcriptional activity of the HIF system. In this schematic, **under Normoxia** hydroxylases utilize oxygen as a co-substrate to catalyzing hydroxylation of a specific proline (P via PHDs) and a specific asparaginyl (N; via FIH) amino acid residues on HIF-α. Proline hydroxylation by PHD marks HIF for degradation (i.e., specifically ubiquitination (**UB)** occurs by VHLE3 ubiquitin ligase (VHL) binding and then rapid degradation by the ubiquitin–proteasome pathway occurs). Hydroxylation at a conserved asparaginyl residue in the HIF-α carboxy-terminal activation domain also blocks interaction with the CBP/p300 transcriptional co-activator required for binding to HIF-α and to DNA. Thus, in normoxia HIF-α is degraded and “Transcriptional Repression” of the HIF system occurs. **Under Hypoxia** PHDs and FIH are inhibited. Without the binding of VHL to non-hydroxylated HIF-α, HIF-α is not degraded. The coactivator CBP/p300 does bind to non-hydroxylated HIF-α that then binds to HIF-β and then to DNA resulting in HIF system “Transcriptional Activation”, which further up-regulates the expression of a large array of target genes. These increased target genes increase protein expressions and cellular processes that result in red blood cell production (e.g., erythropoietin; EPO), angiogenesis (e.g., vascular endothelial growth factor; VEGF), free radical scavenging and stem cell homing and differentiation. GSK360A mimics hypoxia by inhibiting PHDs and producing HIF transcriptional activation and the upregulation of EPO and VEGF target genes and EPO and VEGF protein synthesis. This schematic was modified from Schofield and Ratcliffe, 2004 [71].

There is significant enthusiasm for translational success in the field of pharmacologic preconditioning by targeting the HIF system [2–4, 15–20]. A basic depiction of the HIF system is provided in Fig 1. Briefly, HIF expression consists of inducible HIF-1α and constitutive HIF-1β subunits. HIF-α is a biochemical oxygen (O_2_) sensor and is hydroxylated by a family of hydroxylase enzymes. The inhibition of hydroxylation by directly inhibiting these hydroxylase enzymes produces HIF-1α binding to HIF-1β and results in HIF-transcriptional activation. These events are similar to mild ischemic/hypoxia stress that can be used as a sub-threshold injury preconditioning stimuli that will produce subsequent prolonged cell, tissue and/or organ protection from a subsequent more severe ischemic stress [4].

The initiation of these complex transcript programs through HIF is thought to be crucial for cell adaptation and survival in hypoxic environments. Inhibition of HIF prolyl hydroxylase domain (PHD) enzymes results in activation of HIF and mimics, in large part, the effects of hypoxic/ischemic preconditioning. Endogenously produced erythropoietin (EPO), a transcript and protein up-regulated by HIF signaling, is an essential mediator of ischemic preconditioning and has been identified previously in ischemic tolerant neurons and astrocytes [7, 21–24]. Thus, PHD inhibition could be useful in the treatment of stroke and in other conditions like surgery that can result in post-surgery behavioral/cognitive deficits. Compounds that inhibit PHD, albeit non-selectively, already have demonstrated promising results in preclinical models [17, 20, 25]. HIF-1α adaptive-protective responses have been demonstrated in hypoxic or ischemic conditions in the brain [26], kidney [27] and heart [28]. HIF transcriptional activation includes genes involved in oxygen transport such as EPO [29], angiogenesis such as vascular endothelial growth factor (VEGF) [30], pH regulation [31], energy metabolism such as the glucose transporter -1 (GLUT1) [32], nitric oxide generation [33], and cell motility such as hepatocyte growth factor/scatter factor and its receptor c-Met [34]. More specifically, increased HIF transcriptional activity up-regulates genes and proteins involved in organ protection and cellular proliferation including: erythropoiesis, inflammation and cellular protection (e.g., EPO), angiogenesis (e.g., VEGF), cellular survival and anti-oxidative stress (e.g., heme oxygenase; HO-1), energy metabolism (e.g., pyruvate dehydrogenase kinase; PDK-1) and endothelial nitric oxide synthase [16–18, 35, 36]. Hypoxia or HIF transcriptional activation produces significant protection from focal cerebral ischemia with an increased expression of HIF and its target genes, EPO and VEGF [26]. Increased HIF-1α transcriptional activation has been shown to prevent oxidative cellular death in vitro and ischemic injury in vivo [25].

Fig 1 illustrates how PHD can reduce HIF degradation and increases HIF-induced transcription activity. GSK360A is a novel, orally active PHD inhibitor that increases HIF-1α signaling. The chemical structure and the enzymatic and cellular activity of GSK360A are presented in S1 Fig (as summarized from our previous work; 37). GSK360A is a potent inhibitor of HIF-PHDs (PHD1>PHD2 ≈ PHD3) and capable of activating the HIF-1a pathway in a variety of cell types including neonatal rat ventricular myocytes and H9C2 cells. GSK360A is significantly orally active and has demonstrated significant efficacy in protecting the failing heart [37]. The effects of selective PHD inhibition using GSK360A on brain protection in cerebral ischemia is unknown. Because GSK360A chemically activates the HIF system by its inhibition of PHD, we hypothesized that its administration prior to cerebral ischemia surgery would provide a significant and beneficial brain protective strategy for use prior to surgery. Again, such a pre-surgical protective strategy could be applied to protect the brain under surgical conditions that carry significantly increased risk for post-surgical neurological and cognitive deficits [38–41].

In the present study we examined this “*PHD inhibition—HIF activation and brain protection strategy*” in a rat model of ischemic stroke. A validated dose regimen of GSK360A was administered that activates HIF, increases the downstream expression of transcripts that result in increased protective proteins prior to producing stroke. This GSK360A dose regiment protected the brain from post-stroke injury and significantly reduced neurobehavioral deficits. In doing this, we demonstrate that GSK360A activates HIF transcription as seen in ischemic tolerance to result in brain protection and improved sensory, motor and cognitive outcomes.

## Materials and methods

### Ethics statement

This study was carried out in strict accordance with the recommendations in the Guide for the Care and Use of Laboratory Animals of the National Institutes of Health as well as the ARRIVE (Animal Research: Reporting In Vivo Experiments) guidelines for animal research. The experimental, behavioral and surgical protocol for this research was approved by the SUNY Downstate Institutional Animal Care and Use Committee (IACUC) under IACUC Protocol Number 06-308-10. All surgery was done under deep isoflurane anesthesia. Euthanasia was performed using an overdose of pentobarbital-containing euthanasia solution (e.g., Fatal-Plus) followed by cervical dislocation and decapitation. Blood for plasma, the whole forebrain or ischemic and control forebrain hemispheres, and the kidneys were removed and processed for endpoint measurements as described below. All efforts were made to minimize or eliminate animal suffering.

### Animals

**Fig 2:**
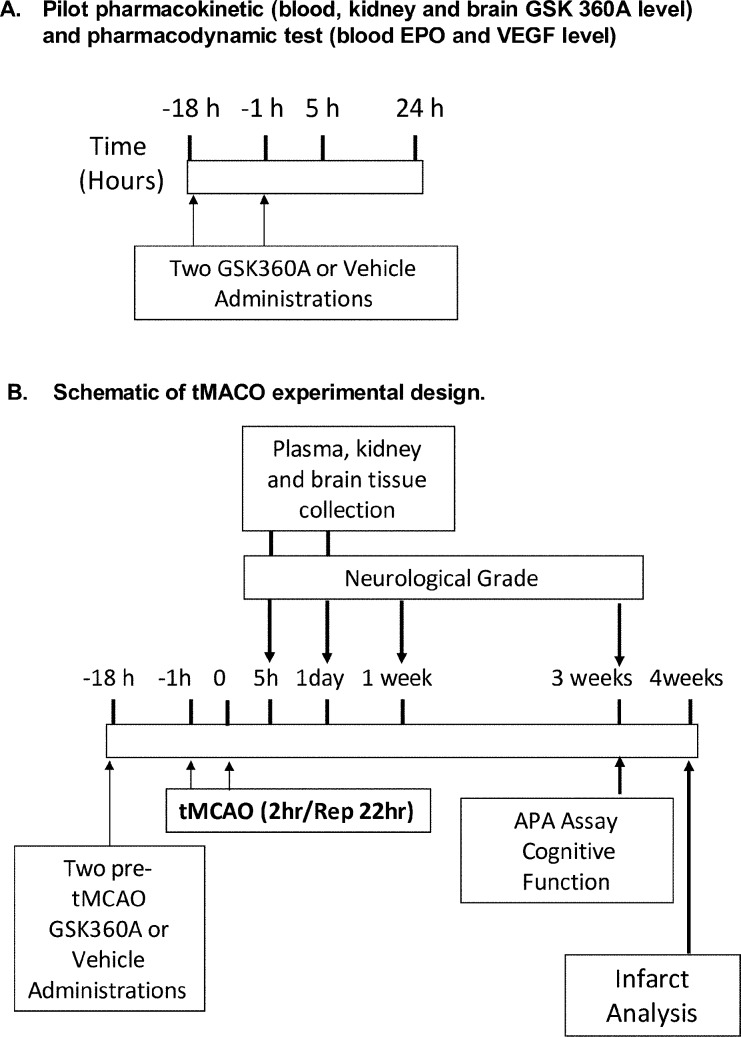
Overall schematic of basic experimental studies and designs. Two-pretreatments of GSK360A (30mg/kg) at 18 hours and 1 hour prior the experiment was administrated orally. (A) Pilot pharmacokinetic and pharmacodynamic tests including plasma, kidney and brain GSK360A levels and blood EPO and VEGF levels were measured 5 and 24 hours after and data were presented in Figs 3 and 4. (B) Ischemic stroke was induced by tMCAO and GSK360A at 18 and 5 hours prior to Stroke that was produced in rats at time 0. The effects of GSK360A on body weight, sensory-motor neurological deficits, cognitive function, biochemical changes on HIF related protein/molecular and brain infarcts were evaluated (Figs 5–10).

Male Sprague-Dawley rats weighing 300 to 350 g from Charles River Laboratories (Wilmington, MA), were housed in pairs under controlled laboratory conditions (e.g., 12 h light/dark cycle, controlled temperature and humidity, ad lib access to food and water). The rats were randomly assigned into different studies and groups that included pilot pharmacokinetic and pharmacodynamic measurements, and transient middle cerebral artery (tMCAO; stroke) experimental measurements of sensory-motor neurological deficits, cognitive performance, brain infarcts/injury and changes in major HIF related transcripts and proteins. In total approximately 100 rats were used for all studies presented here. The detailed experimental design is depicted in Fig 2, and followed the ARRIVE (Animals in Research: Reporting In Vivo Experiments) guidelines (http://journals.plos.org/plosbiology/article?id=10.1371/journal.pbio.1000412).

### Pilot pharmacokinetics—Major organ tissue distribution of oral GSK360A: Plasma, kidney and brain

Analysis of male rat plasma and of brain and kidney homogenate samples collected at 5 and 24 hours after the optimum oral cardioprotective dose (30 mg/Kg; [37]) of GSK360A was performed using liquid chromatography/tandem mass spectrometric (LC/MS/MS) detection. All control group samples were prepared and homogenized prior to any tissues from experimental group samples to avoid cross-contamination. Plasma, brain, and kidney samples (30 μL) were treated with the addition of 120 μL of acetonitrile that contained an appropriate mass spectral internal standard. The resulting mixture was vortex-mixed for 2 min and then centrifuged for 15 min at > 3700 x g. Analytical standards (1.0–10,000 ng/mL) were prepared similarly in rat plasma. Five microliters of the resulting supernatants from the experimental samples and analytical standards were injected onto a 2 x 20 mm, 2.5μ, Synergi Hydro-RP analytical column (Phenomenex, Torrance, CA) using an HTS PAL autosampler (CTC Analytics, Zwingen, Switzerland). The mobile phase consisted of a gradient that transitioned linearly from 90% aqueous formic acid (0.1%) to 100% acetonitrile over 1.7 minutes (750 μL/min flow rate). Mass spectral analysis was conducted with a Sciex API5000 triple-quadrupole mass spectrometer (Applied Biosystems) with negative-ion atmospheric pressure chemical ionization multiple-reaction monitoring. A calibration curve was prepared for GSK360A in rat plasma to confirm sensitivity and linearity of the instrument response, and to quantify GSK360 concentrations in the plasma and tissue samples. The linear analytical range for GSK360A was 1.0 to 10,000 ng/mL. Tissue homogenate concentrations were corrected for GSK360 contained in residual plasma in the tissue.

### Pilot pharmacodynamics—GSK360A effects on plasma EPO and VEGF levels

Circulating levels of EPO and VEGFα proteins were measured at 5 and 24 hours after the optimum dose of GSK360A [37] or Vehicle was administered. Measurements were made using the Mouse/Rat Hypoxia Serum/Plasma Kit (Meso Scale Discovery, Maryland, catalog #K15123C-1). The assay was performed according to manufacturer’s protocol.

### Transient MCAO ischemic stroke surgery and G360A oral administration

Rats were subjected to surgical stroke [42]. Briefly, under isoflurane anesthesia a 3–0 monofilament suture (Ethicon, Somerville, NJ) coated with poly-L-lysine with heat-blunted tip was inserted through left external carotid artery and into the left internal carotid artery and then advanced to occlude the origin of left MCA 18 to 20 mm from the bifurcation of common carotid artery. After 2 hours of left MCAO, rats were re-anesthetized with isoflurane and the intraluminal filament was removed and blood flow to the MCA was restored. Sham groups were subjected to the same procedure but without occluding the MCA. Vehicle (1% Methyl cellulose) or GSK360A (30 mg/kg) was administered to each group of rats by oral gavage (5 ml/kg) at 18 and 5 hours prior to stroke (i.e., based on the pharmacokinetic and pharmacodynamic data; see below). Body temperature was continuously maintained at 37.0 ± 0.5°C using a water heat therapy pump exchange system (HTP-1500, Hallowell EMC, Pittsfield, MA) during surgery and carefully controlled until animals completely recover from anesthesia and display normal motor activity in their living cages following surgery.

### Sensory-motor neurological deficit scores

#### Modified neurologic severity score (mNSS)

The mNSS measurements were performed just prior to surgery and at 5 hours, day 1, and at 1 and 3 weeks after stroke [42, 43] in order to grade post-stroke sensory-motor neurologic deficits. The total score was calculated from individual deficit tests that make up the mNSS which is scaled from 0 to 18 with 0 as normal and the maximal deficit score of18 reflecting the combined sensorimotor, beam balance and reflex-abnormal movement functions [42, 43]. In addition to the total mNSS measure, the Beam Balance component also was analyzed and presented graphically for more detailed investigation into these deficits as done previously [43]. For the Beam Balance Component, rats were placed on a 1 inch-width beam for 60 seconds. A normal response is balance with steady posture for 60 seconds (a score of 0). Deficits are scored if the rat: grasps the side of the beam (a score of 1), hugs the beam and 1 limb falls down from beam (a score of 2), hugs the beam and 2 limb falls off the beam (a score of 3), attempts to balance on beam but falls off from 40–59 seconds (a score of 4), attempts to balance on beam but falls off from 20–39 seconds (a score of 5), or falls off with no attempt to balance or hang on beam in 20 seconds (a score of 6).

#### Foot fault test

Rats were tested at 5 hours, day 1, and at 1 and 3 weeks after stroke for forelimb movement dysfunction while walking on elevated metal grids with randomly missing support bars. The horizontal grids were 85.5×26.5×20 cm3 with a glass enclosure for observation. With each weight-bearing step, the forelimb can fall or slip between the metal support bars, which was recorded as a foot fault. The total number of forelimb steps and the total number of foot faults were recorded. The percentage of forelimb foot faults to total steps that occurred within 2 minutes was calculated [43, 44].

#### Hind limb placing test

The hind limb placing test was also performed at 5 hours, day 1, and at 1 and 3 weeks after stroke. Hind limb placement is a proprioceptive response to limb manipulation. Rats were positioned facing away from the edge of a table with the hind limb contralateral to stroke pulled down and away from the table edge. The ability to retrieve and place the hind limb back onto the table was scored. Immediate and complete limb retrieval was scored 0, delayed (>2 seconds) limb retrieval and/or interspersed flailing was scored 1, and no limb retrieval was scored 2 [43, 45].

#### Active place avoidance learning (APA)

APA as described previously [43] was performed on a single day 3 weeks after stroke and consisted of seven ten-minute trials (each trial separated by 10-minute rest period) on a slowly rotating circular behavioral arena (82-cm diameter metal disc and a 40-cm high transparent wall rotating at 1 revolution/min). The arena was placed in a cylinder-shaped enclosure using a black ceiling-to-floor curtain with prominent visual landmarks inside. Briefly, after a 10 minute exploration period in the arena, the experimenter started running a spot-tracker computer program (BioSignal Group, Brooklyn, NY) which, upon rats entrance into a 60 degree shock zone, would deliver a mild foot shock by passing a constant current (0.3 mA, 60 Hz, 500 ms) through a cable connected to the rat. During each trial, a computer controlled infrared Firewire camera mounted 1.2 m above the arena recorded movement of the rat on the arena. Thus, the rat entrance into the stationary shock zone area on the arena (an invisible 60^**o**^ stationary quadrant on the arena) resulted in negatively reinforcement that needed to be avoided by the rats. The numbers of negative reinforcements (shocks) in each 10 min trial indicates errors in learning performance and were used as the measurement of active place avoidance learning performance. The total path distance the rats traveled in each trail was also measured as the degree of motor activity during these learning trials [46].

### Measurement of brain infarction

To stain the forebrain, 2, 3, 5-triphenyltetrazolium chloride (TTC) was used to measure stroke-induced brain infarctions as described previously [42, 43, 46, 47]. Briefly, brains were removed 4 weeks after stroke and the forebrains were cut into seven 2 mm-thick coronal sections using a brain matrix. These brain sections were immersed in 1% TTC (Sigma-Aldrich, St. Louis, MO) for 20 minutes. After scanning, the image was analyzed and calculated using an image analyzing system IMAGE J 1.37v (NIH, Bethesda, MD) by an observer who was blinded to the study. Original total post-stroke left hemispheric infarcts were determined from the hemispheric loss after 4 weeks, and were normalized to the non-infarcted right hemisphere. Thus, % total left hemisphere infarction over the sections was determined from the degree of hemispheric loss at 4 weeks post-stroke normalized to the right hemisphere calculated as:

Totalinfarct(%)=(righthemisphere–lefthemisphereremainingafterstroke)/(righthemisphere)x100

### Real-time PCR measurements of HIF-related transcripts

To obtain tissue pharmacodynamic data on GSK360A, total RNA was isolated using an RNeasy Mini kit (Qiagen, Valencia, CA), and treated with DNase I (Qiagen) as directed by the manufacturer. cDNA was synthesized by reverse transcription of 2 μg of total RNA using the SuperScript III First-Strand Synthesis kit (Invitrogen, Carlsbad, CA) according to the manufacturer's instructions. Quantitative real-time PCR was performed on StepOnePlus™ Real-Time PCR System (Applied Biosystems Inc, Foster City, CA). Real-time PCR was performed in a 20 μl-reaction mixture containing 10 μl of SYBR Green PCR Master Mix (Applied Biosystems Inc), 2 μl of cDNA, and primer set at 0.3 μM each. The primers were designed using Primer3 software (http://frodo.wi.mit.edu/cgi-bin/primer3/primer3_www.cgi) as following: 18S rRNA, ACC GCG GTT CTA TTT TGT TG and CCC TCT TAA TCA TGG CCT CA; EPO, CAC GAA GCC ATG AAG ACA GA and GGC TGT TGC CAG TGG TAT TT and VEGF, GCC CTG AGT CAA GAG GAC AG and CAG GCT CCT GAT TCT TCC AG. The amplification consisted of one cycle at 95°C for 10 min, followed by 40 cycles at 95°C for 15 s and at 60°C for 60 s. Serial dilutions of sample cDNA were used to obtain a calibration curve. The individual target was quantified by determination of the cycle threshold (Ct) and by use of calibration curves. At the end of amplification, the specificity of the PCR was confirmed by melting temperature. The relative amount of the target was normalized with 18S rRNA and expressed as arbitrary units [48].

### Statistical analysis

All data were expressed as means ± SEM. For comparison between multiple groups, statistical analyses were performed using two-way analysis of variances followed by post hoc analyses using Bonferroni procedure for multiple comparisons. For comparison between only two groups, t-tests were used. Differences between groups were considered significant if p< 0.05. Although parametric statistics were appropriate and used for these data, non-parametric ANOVAs or Mann-Whitney U-tests were also performed and both produced identical results.

## Results

### GSK360A pharmacokinetics (Pilot PK) and organ penetration

**Fig 3:**
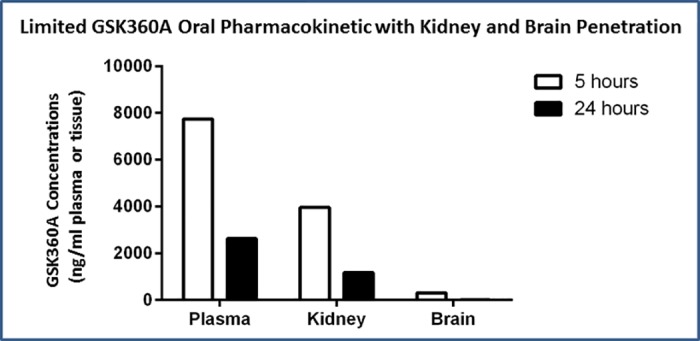
The blood plasma, kidney, and brain concentration levels (ng/ml) of GSK360A presented for 5 and 24 hours after GSK360A (30 mg/kg) was administered orally by gavage. Adequate plasma and peripheral end-organ levels were achieved as also cross-validated by our previous plasma data (37). N = 2 rats per measurement; corroborated by previous data [37].

In order to test our hypothesis, we first needed to better characterize the optimum GSK360A “dose-regimen” used previously in heart failure [37] for pharmacokinetic and pharmacodynamic changes over time to validate its use in the present shorter-term stroke study. Pharmacokinetic distributions in major organ tissues (i.e., from plasma to kidney and brain penetration) and pharmacodynamic plasma protein changes (e.g., HIF-driven transcripts; EPO and VEGF) were measured. Thus, we collected the data that demonstrated GSK360A produced adequate HIF transcriptional activation with appropriate timing to cover our experimental stroke condition both prior to and at the time of stroke surgery. After an oral dose of 30 mg/kg of GSK360, the drug was readily absorbed into the circulation and the drug level in plasma reached 7734 ng/ml (22.2 μM) 5 hours after dosing and 2629 ng/ml (7.5 μM) 24 hours after dosing (Fig 3), as corroborated by our previous PK data (37). The compound was distributed to kidney with concentrations of 3956 ng/ml (about 11.4 μM) and 1172 ng/ml (about 3.7 μM) at 5 hours and 24 hours after dosing, respectively. However, exposure in the brain was much less than plasma and kidney. Brain levels only reached about 300 ng/ml (about 1 μM) and 40 ng/ml (about 0.1 μM) 5 hours and 24 hours after dosing, respectively (Fig 3). These data suggest that although oral administration of GSK360A results in significant exposure in peripheral tissues such as plasma and kidney, its brain concentration is much less. Despite this, localized brain effects by GSK360A cannot be ruled out due to its potency (PHD IC50s ~ 10–100 nM; See S1 Fig) as will be discussed below.

### GSK360A pharmacodynamics (Pilot PD): plasma EPO and VEGF protein levels

**Fig 4:**
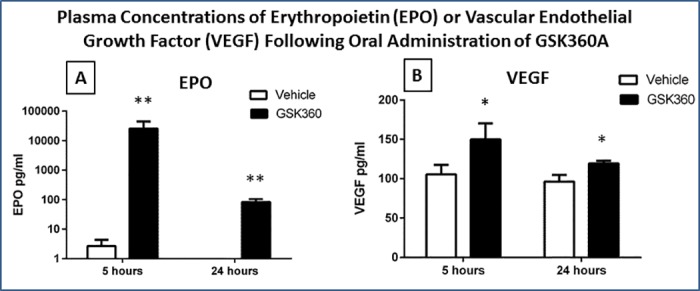
(**A)** The blood plasma levels of EPO 5 hours and 24 hours after 30 mg/kg, p.o. GSK360A administration is presented. The GSK360A treated group had significantly higher levels of EPO compared to vehicle group even at 24 hours after administration (e.g., >80-fold over the vehicle group for both time points). (**B)** The blood plasma levels of VEGF 5 hours and 24 hours after GSK360A administration is presented. GSK360A treated group had significantly higher levels of VEGF at both time points compared to vehicle group. N = 5 rats per group. Two-way ANOVA test followed by post hoc analysis using the Bonferroni procedure for multiple comparisons; *p < 0.05 and **p < 0.001 when compared with vehicle group.

The oral administration of GSK360A significantly upregulated HIF-1α target EPO and VEGF levels in rat plasma (Fig 4). A remarkable increase of plasma EPO level produced by GSK360A was observed both at 5 and 24 hours (2–3 orders of magnitude; p<0.001). A significant increase (20–50%) in VEGF produced by GSK360A was observed at 5 and 24 hours after administration (P < 0.05). The results of data in Figs 3 and 4 provided the basis of our decision to dose twice (at 18 and 5 hours prior to stroke) to provide the desired pharmacokinetic and pharmacodynamic consequence of GSK360A administration at the time of stroke. Thus, since we verified adequate GSK360A plasma levels and end-organ levels (Fig 3) and GSK360A-induced HIF end-organ pharmacodynamic changes (Fig 4) under these conditions (i.e., as cross-validated with our previous data; 37), we then were satisfied that the 30 mg/kg GSK360A exposure was adequate for testing efficacy in ischemic stroke.

### Effects of stroke and G360A oral administration on body weight

**Fig 5:**
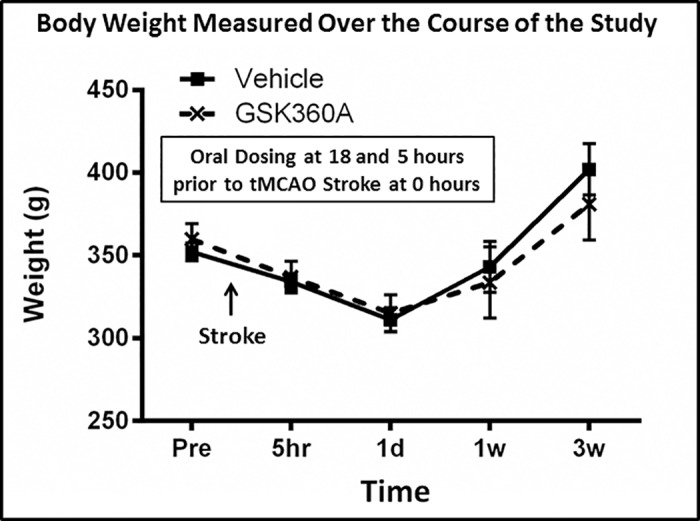
Body weight was measured over the course of the study. Thus, 24 hours prior to tMCAO Stroke body weight was measured and represents the “Pre” measurement. This was followed by the oral (gavage) administration of 30mg/kg GSK360A at 18 and 5 hours prior to Stroke that was produced in rats at time 0. Then body weight was measured at 5 hours, 1 day, 1 week and 3 weeks after stroke. No significant differences in body weight were observed between the vehicle and the GSK360A treated groups over the course of study. N = 8 rats per group. Two-way ANOVA test followed by post hoc analysis using the Bonferroni procedure for multiple comparisons. There was no difference in body weight over time (p = 0.7064) between vehicle and GSK360A groups.

Animal weights were measured one day before surgery (pre-stroke) and at 5 hours, 1 day, 1 week and 3 weeks after stroke. Stroke produced a similar non-significant transient decrease in body weight in both vehicle and GSK360A treated groups (e.g., similar decreases of 11.6% in vehicle and 12.4% in GSK360A groups; p > 0.05) at 1 day after stroke. There was no difference in body weight over time (p = 0.7064) between vehicle and GSK360A (Fig 5).

### Effects of G360A on neurological deficits after stroke

**Fig 6:**
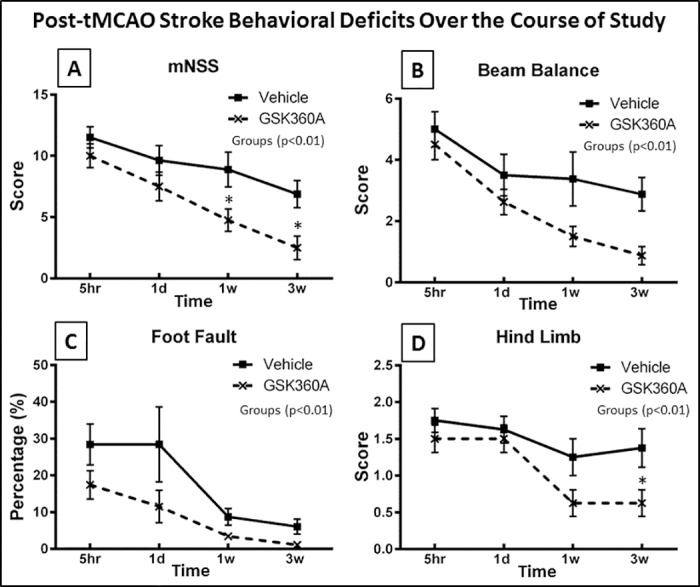
The effects of GSK360A on **(A)** mNSS, **(B)** beam balance, **(C)** foot fault and **(D)** hind limb performance at 5 hours, 1 day, 1 week and 3 weeks after stroke. GSK360A treatment significantly reduced post-stroke deficits in all tests (ANOVA group factor for all tests p < 0.01). Additional analysis following up on the significant (p < 0.01) group by trial interaction indicated that mNSS was significantly decreased compared to vehicle treatment at 1 and 3 weeks post-tMCAO stroke. Beam Balance performance, a component of the mNSS was similarly improved by GSK360A treatment. GSK 360A also produced a significant overall improvement in foot fault performance. Finally, GSK360A treatment produced a significant overall improvement in hind limb deficit, and following up on the significant (p < 0.01) group by time interaction resulted in identifying hind limb deficit score reduced compared to vehicle at 3 weeks after stroke. N = 8 rats per group. Two-Way ANOVA, followed by post hoc analysis using the Bonferroni procedure for multiple comparisons; *p < 0.05 when compared with vehicle groups.

Stroke induces significant neurological deficits with some gradual improvement/recovery over time as described previously [43]. In the overall mNSS and some of the individual mNSS behavioral components studied there were significant differences between vehicle and GSK360A groups after stroke (Fig 6). Results of the mNSS two-way ANOVA indicated that the Group factor (p<0.01) and the Group x Time interaction Factor was significant (p<0.0001) for mNSS. This was also shown for mNSS motor (p<0.01), sensory (p<0.05) and beam balance (p<0.01) components as segregated previously [43]. Post-hoc test follow-up using the Bonferroni procedure showed that the stroke-induced increases in overall mNSS were reduced 46.5% and 63.6% (p<0.05) by GSK360A at 1 and 3 weeks, respectively, post-stroke compared to vehicle. Moreover, the group factor was significant (p < 0.01) indicating that GSK360A treatment also improved overall performance compared to vehicle administration in Beam balance, foot fault and hind limb tests. In the Hind Limb test, the group x time interaction factor also was significant (p<0.05) and a follow-up using the Bonferroni procedure identified that GSK360A provided significant improvement at 3 weeks post-stroke compared to vehicle (p<0.05, Fig 6).

### Effects of G360A on cognitive function after stroke

**Fig 7:**
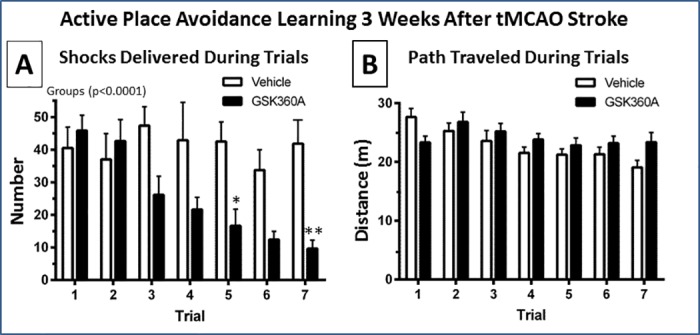
Effect of GSK360A on APA learning performance 3 weeks after tMCAO stroke. **(A)** The Two-Way ANOVA indicated that the group and group x trial interaction effects were significant (p < 0.01). Thus, looking at the interaction of groups over trials, the GSK360A treated group received significantly less shocks (i.e., made less errors in avoiding the shock quadrant) over trials (p < 0.01). Specifically, the GSK360A treated group received significantly less shocks in trial 5 and trial 7. **(B)** The effect of GSK360A was not due to differences in rat movements during the trials, as there was no significant difference in distance traveled per trial between the two groups. N = 8 rats per group. Two-Way ANOVA, followed by post hoc analysis using the Bonferroni procedure for multiple comparisons; *p < 0.05 and **p < 0.01 when compared with vehicle groups.

APA learning performance was used to evaluate cognitive learning performance (Fig 7). Stroke resulted in a significant impairment in APA performance (e.g., the vehicle group deficit in this present study was similar to that described previously; [43]). Rats treated with GSK360A exhibited a significantly decreased overall number of shocks compared to vehicle group (e.g., group factor p<0.0001). The group x trials interaction factor was also significant (p < 0.01) and follow-up analysis indicated GSK360A treatment resulted in significantly reduced shocks on trials 5 and 7 (p<0.05 and p<0.01, 60 and 75%, respectively) compared to vehicle treatment. Thus, GSK360A treatment decreased the number of shocks (e.g., errors) over trials, demonstrating an improved ability of rats to learn to avoid the shocked quadrant of the rotating arena. There was no significant difference in the distance traveled during the APA trials between the vehicle and GSK360A groups. Thus, since the total distance during each trial is similar in both groups, motor deficit differences between the groups do not explain the GSK360A improvement in cognitive performance (Fig 7).

### GSK360A effects on plasma EPO and VEGF protein levels after stroke

**Fig 8:**
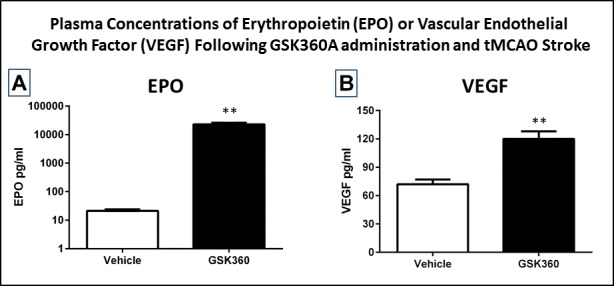
GSK360A treatment significantly increased both **(A)** plasma EPO and **(B)** plasma VEGF levels 24 hours after stroke. The increase in EPO 24 hours after stroke was greater than that produced by the same dose without stroke suggesting a more prolonged increase than without stroke as shown in Fig 4. N = 8–10 rats per group. t-test; **p < 0.01 when compared with vehicle groups.

There were significant differences between vehicle and GSK360A for EPO and VEGF levels 24 hours after tMCAO stroke (p<0.01) (Fig 8). The GSK360-induced increase in EPO was higher 24 hour post-stroke suggesting a more prolonged increase (Fig 8) then that observed without stroke (Fig 4). VEGF increases were similar with or without stroke (compare Figs 4 and 8). Overall, these data in the presence of stroke are consistent with the pilot PK and PD data described above here and previously (37).

### GSK360A effects on kidney EPO mRNA and brain VEGF mRNA levels after stroke

**Fig 9:**
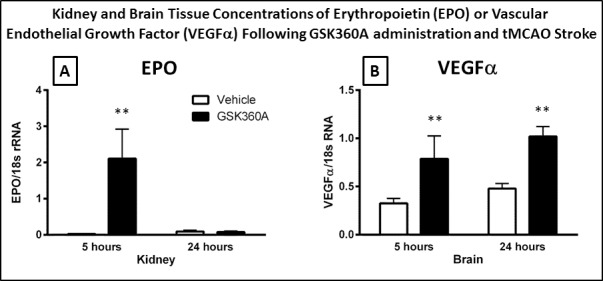
GSK360A increased kidney EPO and brain VEGF mRNA after stroke. (A) EPO mRNA in the kidney was significantly increased by GSK360A at 5 hours post-stroke compared to vehicle group. Levels of EPO mRNA returned to vehicle treated levels by 24 hours post-stroke.(B) VEGF mRNA in the brain ischemic hemisphere (above) and in the non-ischemic hemisphere (not shown) was increased by GSK360A at 5 hours and 24 hours post-stroke compared to vehicle group (p = 0.002). N = 5 rats per group. Two-Way ANOVA test, followed by post hoc analyses using the Bonferroni procedure for multiple comparisons; **p < 0.01 when compared with vehicle groups.

In the kidney, GSK360A dramatically increased EPO mRNA early post-stroke as compared to vehicle (e.g., group by time interaction factor was significant; p<0.05) (Fig 9). Specifically, GSK360A increased kidney EPO mRNA up to 80-fold (p<0.01) by 5 hours post-tMCAO stroke (i.e., an early induction of EPO mRNA by GSK360A) (Fig 9) that appears to contribute to the prolonged elevation of plasma EPO protein as shown in Fig 8. There was a trend for GSK360A to increase 5 hour PDK-1 and also a trend for GSK360A to increase in both vehicle and GSK360A post-stroke HO-1 (not significant; data not shown). No differences in kidney mRNA expression for VEGF, EGL nine homolog 2 or Stromal cell-derived factor 1 were observed (not significant; data not shown).

In the brain ischemic hemisphere, GSK360A significantly increased VEGF subunit mRNA (two-way ANOVA overall group factor was significant; p<0.01). At 24 hours post-stroke VEGF mRNA increased 213% in the ischemic hemisphere compare to vehicle group (two-way ANOVA group x time interaction effects and post hoc Bonferroni test p<0.01, Fig 9). A similar increased VEGF subunit mRNA effect was also observed in the non-ischemic hemisphere (data not shown). Although there was a trend to increase in both vehicle and GSK360A groups the post-stroke HO 1, and stromal cell-derived factor 1 and EPO in both hemispheres (not significant; data not shown), there were also no significant brain changes in the mRNA expression for PDK-1 or EGL nine homolog 2 in either brain hemisphere following stroke for either group (not significant; data not shown). Thus, orally administered GSK360A significantly upregulated brain VEGF but did not significantly upregulate brain EPO. Apparently, the brain protective effects of EPO under these conditions would be due to the remarkably increased plasma EPO protein produced by GSK360A.

### G360A effects on brain infarctions after transient MCAO

**Fig 10:**
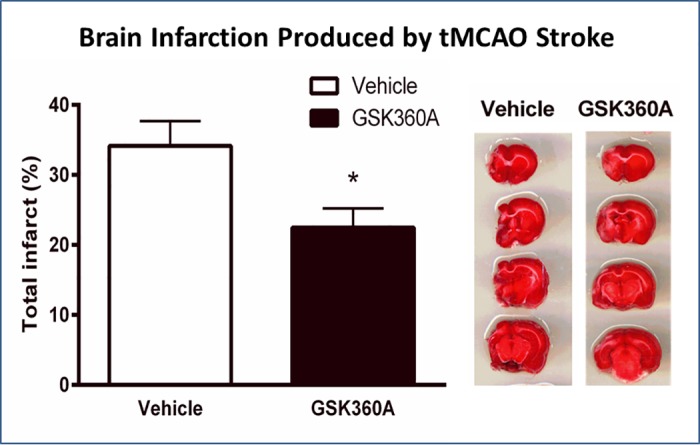
TTC staining of forebrain 2 mm thick sections conducted 4 weeks after tMCAO stroke (1 week after APA testing). GSK360A treatment resulted in a 30% decrease in hemispheric loss reflecting the decreased infarct size compared to vehicle treatment. N = 10 rats per group. t- test; *p < 0.05 when compared with vehicle group.

Four weeks after tMCAO stroke the brain infarctions were measured using TTC staining as described previously [48, 49]. GSK360A had decreased the hemispheric loss corresponding to the original infarct size by 30% (p<0.05, Fig 10).

## Discussion

The HIF transcription system is activated by low oxygen tension and controls a diverse range of cellular processes including angiogenesis, erythropoiesis and cellular metabolism targeted at increasing oxygen delivery to tissues. Prolyl hydroxylation regulation of HIF has been well-studied. Evidence from a number of laboratories supports the notion that HIF PHD inhibition can improve histological and functional outcomes in ischemic and hemorrhagic stroke models [50–52]. Our major finding in this present study is that oral pretreatment of PHD inhibition with GSK360A improved sensory, motor and cognitive functioning and reduced brain injury after tMCAO stroke. These effects were associated with remarkable increases in circulating EPO and significantly increased VEGF levels.

Here we utilized pretreatment with GSK360A at 18 and 5 hours before tMCAO stroke to test our hypothesis. The dose and times we selected were based on the pharmacokinetics and pharmacodynamics data presented here and from our previous in vivo experience [37]. The effects of pretreatment, however, were persistent, and improvements in neurobehavioral effects were maintained at 3 weeks post-stroke. Several neurobehavioral measurements and analyses were made including: mNSS, beam balance, foot fault and hind limb. The data demonstrates that GSK360A significantly improves functional outcome after stroke. Moreover, the data indicates that GSK360A improves/protects cognition and decreases learning deficits due to brain injury in APA. For APA, rats require neither deprivation nor pre-training but rapidly learn to avoid “negatively reinforcing mild electrical shocks” by paying attention to their position relative to distal room landmarks on a continuously rotating arena [53]. This method has been identified as a very sensitive measurement of mild traumatic brain injury [54] and stroke [43]. In the present study, we show this test’s sensitivity to brain protection using GSK360A. Using this cognitive assay, the total travel distance during each trial is measured in order to compare and exclude the influence of altered motor function on stroke-induced deficits in learning. There was no difference in distance traveled over learning trials between the GSK360A and vehicle treated groups strongly suggesting that GSK360A cognitive protection/improvement is not due to differences in ability to move in the APA environment. Stroke-induced cognitive impairment in the rat is similar to vascular cognitive impairment or vascular dementia observed in man due to stroke [13, 43].

TTC staining has been used to measure brain injury in this study. Four weeks after stroke, the original infarct has been calculated as brain loss in TTC staining and the results has shown that GSK360A significantly decreased infarctions as compared to the vehicle group, indicating a significant protection of ischemic brain tissue. Here we show this for GSK360A both functionally (e.g., dramatic improvements in a profile of neurobehavioral end-points) and histologically (e.g., reduced brain infarction). This is the first study demonstrating that pretreatment with a selective HIF PHD inhibitor provides effective, long-term brain and behavioral protection from stroke. These results essentially support our hypothesis that pretreatment can protect the brain from brain insults that could occur during surgery. Data in mouse stroke indicates that PHD inhibition in stroke is efficacious in part due to protection of the blood brain barrier and neuronal protection [52].

GSK360A is a selective PHD inhibitor and contributes to HIF-1α stabilization and EPO production [37]. GSK360A selectively inhibits the function of PHD which normally hydroxylates HIF-1α and targets it for degradation (Fig 1). Under PHD inhibition (i.e., similar to hypoxic conditions), the post-translational modification of HIF-1α is inhibited and stabilized, which promotes the transcriptional activation of genes, including EPO and VEGF [55]. Here we found that GSK360A induced EPO production well above normal limits in plasma that persisted 24 hours after stroke. EPO is a cytokine produced by fibroblast-like cells in the kidney. Here, kidney EPO mRNA expression was also observed to increase much earlier (i.e., 5 hours after tMCAO stroke). Thus the early increased kidney EPO mRNA might be responsible for the persistently increased EPO released into plasma 24 hours later. Here we show that increased circulating EPO can be responsible for GSK360A brain and behavioral protection.

GSK360A induced brain VEGF mRNA expression and VEGF release into the circulation. This effect of GSK360A pretreatment was significant 24 hours after stroke. VEGF is the most potent in vivo promoter of angiogenesis. The main angiogenesis-related functions of VEGF include promoting endothelial cell survival, inducing their proliferation and enhancing their migration [56]. In addition to its crucial role in angiogenesis, VEGF may be involved in several other processes in the central nervous system, such as neural cell development, neurotrophic and neuroprotective effects [57, 58]. A number of studies assessing the VEGF expression in brain tissue following an acute ischemic stroke were performed on animal models [59]. VEGF might have both neuronal and glial protective effects in addition to angiogenetic properties to mitigate the effects of ischemia [60]. For example, inhibition of PHD can induce rapid and transient expression of HIF-1α and downstream targets of HIF including VEGF in endothelial and smooth muscle cells and induced endothelial cell-specific proliferation [61]. Thus, GSK360A-induced increased VEGF levels might improve histological and functional outcome from stroke through multiple mechanisms (e.g., exerting acute brain protection as well as later effects to increase neuronal survival and angiogenesis) [62]. GSK360A activation of the HIF pathway through PHD and the resulting secretion of the brain protective proteins (e.g., EPO and VEGF) that represent mixed mechanisms of action in the treatment of ischemic disease. Thus, EPO and VEGF apparently induce a systematic bias toward protection, repair and functional recovery in ischemia [63].

HIF is a heterodimeric transcription factor that is stabilized by hypoxia or growth factors following stroke [18, 64]. Stabilized HIF can translocate to the nucleus to bind to hypoxia response elements in a cassette of genes that mediate adaptive responses to ischemia. Many years of research has focused on the roles of HIF PHD inhibition and HIF-dependent and HIF-independent pathways in neuroprotection. However, we can also expect the cellular and molecular effects of HIF PHD inhibitors on mechanisms of brain restoration/regeneration to be significant. Thus, PHD inhibition stimulation of recovery in chronic stroke also needs to be investigated in the future. In fact, some data in the present work suggests that function might improve more rapidly following GSK360A administration (e.g., note these trends in Fig 7). Recently, it has been shown that HIF-1α binding to the Epac1 promoter recruits hematopoietic stem cells to the ischemic brain following stroke [65]. Also, HIF-1α modified or hypoxia conditioned mesenchymal or progenitor stem cells are an effective means of promoting their regenerative capability and therapeutic potential for the treatment of ischemic stroke. These HIF-mediated effects are mediated at least in part through VEGF/PI3K/Akt/Foxo1 cell protective pathways and involve angiogenesis [66–69] and may be involved in the brain and behavioral effects demonstrated here.

GSK360A oral activity and ability to penetrate and sequester in organs like the kidney is significant. One interesting issue in the present study is the poor degree of brain penetration for GSK360A. Although it can penetrate into the brain, its penetration is very low and apparently its brain protection efficacy might be due, at least in large part, to peripheral EPO levels that are achieved following GSK360A. It is important to mention that 1 uM of GSK360A exposure in brain might still result in a local brain effect as HIF has direct cell/tissue protective mechanisms that can be independent of EPO such as those on apoptosis factors—BCL2/Bax, and SNIP3 [36]. Alternately, a clear increase in brain mRNA for VEGF was observed and thus expected changes in the brain HIF system resulted GSK360A that could protect and restore brain functioning as discussed above. These differential effects on HIF transcriptional changes are difficult to understand but might be associated with brain cell types exposed to GSK360A during brain injury [70]. For example, astrocytes having a propensity to produce VEGF might be highly exposed due to early ischemic injury to the blood brain barrier. Clearly, in the future we need to focus on the study of PHD inhibitors with improved brain penetration and the results of their effects on the brain HIF system. This should be characterized and compared for brain protection and also for their potential efficacy to: [1] protect the brain when administered early after stroke has occurred (i.e., post-stroke rather than pre-stroke treatments), and [2] induce restoration of impaired brain functioning later after stroke brain injury has already occurred.

## Conclusions

Here the oral pre-stroke administration of the HIF prolyl hydroxylase inhibitor GSK360A significantly increased pharmacodynamic indices of HIF transactivation and provides brain protection and long-term functional improvements in post-stroke neurological outcome. Beneficial effects to reduce stroke-induced cognitive impairment were significant. Thus, GSK360A use prior to surgery as a brain prophylactic strategy can be expected to reduce the probability of post-surgical cognitive decline. Since it can protect the heart as well, such a pre-heart or pre-brain surgery treatment is especially appealing for GSK360A.

## Supporting information

S1 Fig The chemical structure and the enzymatic and cellular activity of GSK360A. Chemical structure of GSK360A, N-{[1-(2-cyclopropylethyl)-6-fluoro-4-hydroxy-2- oxo-1, 2-dihydro-3-quinolinyl] carbonyl} glycine is shown at the left. GSK360A one-half maximal inhibitory concentrations (i.e., pIC50 in μM) for the 3 isolated prolyl hydroxylase (PDH) enzyme isoforms (i.e., 1, 2 and 3) are listed. GSK360A is a potent inhibitor of HIF-PHDs (PHD1>PHD2 ≈ PHD3) with pIC50 values of 8.0, 7.0 and 6.9 respectively. In Hep3B cells, GSK360A increased cellular EPO by 13-fold at 3 μM and VEGF by 3-fold at 1 μM. These data are summarized from our previous work [37] in order to provide background information on GSK360A biological activity.
(TIF)

## Abbreviations

APA: active place avoidance
EPO: erythropoietin
HIF: hypoxia-inducible factor
IP: ischemic preconditioning
IT: ischemic tolerance
mNSS: modified neurological severity score
PHD: prolyl 4-hydroxylase
tMCAO: transient middle cerebral artery occlusion stroke
TTC: triphenyltetrazolium chloride
VEGF: Vascular Endothelial Growth Factor
