# Supplementary figures and images for: The prolyl 4-hydroxylase inhibitor GSK360A decreases post-stroke brain injury and sensory, motor, and cognitive behavioral deficits
Source: PLoS One. 2017 Sep 7;12(9):e0184049. doi: 10.1371/journal.pone.0184049 (PMC5589177; doi:10.1371/journal.pone.0184049)

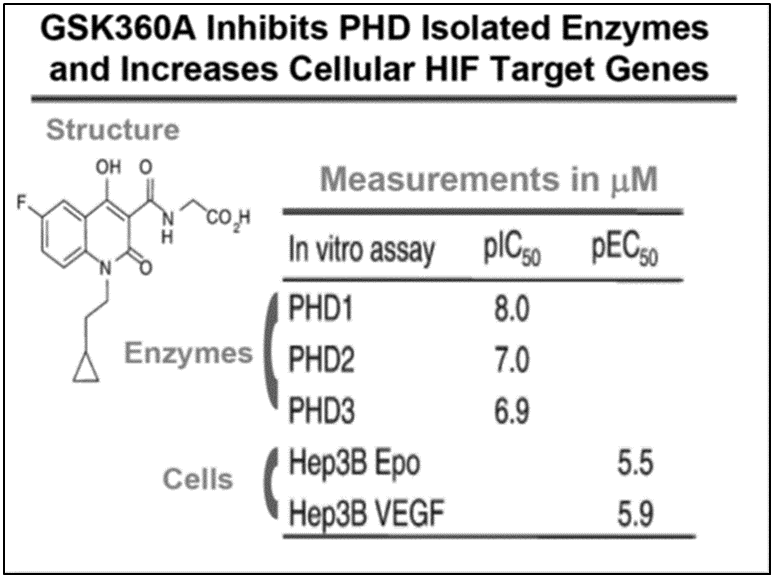

Supplement: S1 Fig — Chemical structure of GSK360A, N-{[1-(2-cyclopropylethyl)-6-fluoro-4-hydroxy-2- oxo-1, 2-dihydro-3-quinolinyl] carbonyl} glycine is shown at the left. GSK360A one-half maximal inhibitory concentrations (i.e., pIC50 in μM) for the 3 isolated prolyl hydroxylase (PDH) enzyme isoforms (i.e., 1, 2 and 3) are listed. GSK360A is a potent inhibitor of HIF-PHDs (PHD1>PHD2 ≈ PHD3) with pIC50 values of 8.0, 7.0 and 6.9 respectively. In Hep3B cells, GSK360A increased cellular EPO by 13-fold at 3 μM and VEGF by 3-fold at 1 μM. These data are summarized from our previous work [37] in order to provide background information on GSK360A biological activity. (TIF) [file pone.0184049.s001.tif]
